# Compression and Immobilization Principal Factors in Surgery

**Published:** 1880-01

**Authors:** George Halsted Boyland

**Affiliations:** Baltimore, Md.


### COMPRESSION AND IMMOBILIZATION PRINCIPAL FACTORS IN SURGERY.

By GEORGE HALSTED BOYLAND, A.M., M.D., ETC., BALTIMORE, MD.

In the general treatment of, perhaps, the majority of surgical diseases, compression and immobilization are often either entirely overlooked or regarded as matters of only secondary consideration, when they are in reality the pivot upon which surgical therapy to a very great extent turns. What would become of certain arterioles to which the ligature and cautery are alike inapplicable, were it not that a ready means of arresting the blood flow presents itself in compression? Or how could the edges of often large wounds, to which, for scientific reasons, it is deemed best not to lay on sutures, be kept together were it not for compression? In fact, compression is to a great degree taking the place of the suture altogether, as witness after surgical operations, the bringing into contact of wound surfaces by means of diachylon.

With the elastic stocking for varicose veins, with adhesive plaster, the compress and roller bandages in all minor and some major cases, sufficient means are at hand to exercise the necessary compression in general surgery.

There are instances, however, in which these do not suffice, and where a long continued mechanical and, so to to speak, foreign compression is required. Of the various methods suggested to this end, the employment of air or water seems to merit the first place. Dr. Chassagny employed this method very ingeniously, the principle of which consists in applying a soft, even pressure to surfaces by means of a thin India-rubber sack, gradually filled with water or air, protected in the upper side by a fixed covering, and resting on the under side upon the organ to be compressed, in such a manner as to fit itself exactly to any unevennesses that may exist. It is desirable to compress a circumscribed surface, as a breast, a tumor, an aneurism, then a solid envelope of metal or papier mache ought to be prepared and fastened at its base with proper bands, the whole fitting the tumor exactly. Within this envelope the India-rubber sock is placed, the tube of which is left to project so that the sack can be inflated by air or filled with water. To exercise circular compression upon the body, upon a limb in its continuity, upon a joint, the following procedure is proposed: Let a circular envelope of the shape of the limb be made of metal, zinc or tin, to open and shut by small hinges; the limb is first wrapped in the India-rubber sack, made very long, of course, and to correspond as nearly as possible to the shape of the limb, and then the metallic envelope is applied over it; the two tubes in this instance projecting. The metallic envelope is closed at either end by a cushion, encircling the limb in such a manner as to prevent the India-rubber sack from spreading out in any other direction than the circumference of the limb. Instead of this, steel bands, with stiffening and linen, after the manner of corsets, might be used in making the outer envelope.

The latter mode, the author very properly hints, might be unsuitable in fractures. Indeed, it is altogether too complicated to be used as a regular surgical appliance, and is open to the very great objection that the steel bands, being placed at intervals, would exercise more compression in those places where they were, and thus defeat the main object, viz, evenness, and, as much as possible, softness.

The inconvenience attending lacing and unlacing would be of minor importance, although that it not without objection as to immobilization, and immobilization is the counterpart of compression. In fractures it is the *sine qua non*, but immobilization claims a position in the treatment of almost all wounds, whether of flesh or bone, especially in wounds of the extremities; even in such insignificant matters as finger wounds, are better, quicker and surer results obtained by immobilization.

Compression and immobilization bear the same relation to the art of surgery that anatomy and pathological anatomy bear to its science. As regards the treatment of the mamma on this principle, the metallic envelope and India-rubber bag would be applied by straps, which would have to pass around the body and neck to keep the apparatus in place—being made in buckles at the base of the metallic envelope. In the same manner can methodic compression be carried out on any tumor; but with aneurisms the system would be the one, *par excellence*, as causing much less pain and being more effective than digital compression. Water produces a milder, and, on account of its weight, a somewhat more powerful compression, and has the advantage over air that it can be turned into an irrigator at will.

Œdema on the extremities does not occur if the apparatus is evenly applied. Compression and immobilization by means of water and air, undoubtedly are of value in some inflammatory and traumatic affections of the extremities, but in fractures this method stands far behind the plaster of Paris bandage. The fact is often lost sight of, that, after long compression has been exercised, a physiological immobilization of the muscles takes place (a kind of paralytic condition,) which prevents shortening and angular positions of fragments by muscular pressure just as well as mechanical immobilization; which, however, must not be neglected, and is better accomplished either by the plaster of Paris bandage or thick pasteboard, moistened and bent on to the limb so as to take its shape when dry, than by splints of any kind. In fractures of the patella, immobilization and compression are best attained by means of the rubber sack and metallic envelope, as the sack, filling any hollows that exist either above or below the fracture, would thus continually press the fragments together.

The author of the metallic covering and rubber bag dressing proposes for amputation stumps the same method, with the simple modification of having two tubes, one communicating without for purposes of inflation, the other communicating directly with the wound surface for drainage. Experience has shown that compresses, roller band dages, and adhesive plaster, are all sufficient here, and are best because they are simplest.

As to the clinical facts with the metallic covering and rubber sack they are indeed few; nevertheless, they do warrant its application in similar cases. Dr. Chassagny used it in six cases of mastitis with very good results; the pains ceased, and swelling and suppuration diminished at once. In two cases of hydrarthrus genu the extravasation disappeared in the first twenty-four hours, and complete healing took place in fourteen days. Like results in a case of very painful gouty inflammation of the knee joint, where, after application of the method, great relief followed, while pain and tumor disappeared in twelve hours. A large extravasation of blood in the region of the Achilles tendon, and caused by a blow, was entirely resorbed the next day, on which the patient was able to go about with a cane. These statistics are certainly gratifying, and invite a more general introduction of the method in certain cases to which it is pre-eminently suited.

In all wounds that we wish to heal per primam, immobilization is desirable, continued compression absolutely necessary; continued, in distinction from the compression of operative surgery, whether digital, by tourniquet or Esmarch's bandage. The inconvenience and needless pain of sutures may often be spared by basing our treatment on these fundamental principles. If, however, it is deemed best to apply sutures, compression should be exercised as if they were not there. In a case that came under our own observation, of long, deep flesh wound, extending from the acromion to the external condyle of the humerus, immobilization and compression were obtained by adhesive plaster strips and roller bandages; no sutures were applied, although if ever necessary they were then. This article does not advance any new theory; does not claim any recent discovery, but simply is intended to rescue from indifference matters that seemed to be passed over lightly, or taken as a matter of course, in the routine of surgical practice, when compression and immobilization not only seem to us principal factors in but the very foundation upon which both ancient and modern surgical art is built.—*The Practitioner.*

